# The impact of fornix lesions in rats on spatial learning tasks sensitive to anterior thalamic and hippocampal damage

**DOI:** 10.1016/j.bbr.2014.10.016

**Published:** 2015-02-01

**Authors:** Julie R. Dumont, Eman Amin, Nicholas F. Wright, Christopher M. Dillingham, John P. Aggleton

**Affiliations:** School of Psychology, Cardiff University, 70 Park Place, Cardiff CF10 3AT, Wales, UK

**Keywords:** Biconditional learning, Cognitive map, Configural learning, Hippocampus, Incidental learning, Thalamus

## Abstract

The present study sought to understand how the hippocampus and anterior thalamic nuclei are conjointly required for spatial learning by examining the impact of cutting a major tract (the fornix) that interconnects these two sites. The initial experiments examined the consequences of fornix lesions in rats on spatial biconditional discrimination learning. The rationale arose from previous findings showing that fornix lesions spare the learning of spatial biconditional tasks, despite the same task being highly sensitive to both hippocampal and anterior thalamic nuclei lesions. In the present study, fornix lesions only delayed acquisition of the spatial biconditional task, pointing to additional contributions from non-fornical routes linking the hippocampus with the anterior thalamic nuclei. The same fornix lesions spared the learning of an analogous nonspatial biconditional task that used local contextual cues. Subsequent tests, including T-maze place alternation, place learning in a cross-maze, and a go/no-go place discrimination, highlighted the impact of fornix lesions when distal spatial information is used flexibly to guide behaviour. The final experiment examined the ability to learn incidentally the spatial features of a square water-maze that had differently patterned walls. Fornix lesions disrupted performance but did not stop the rats from distinguishing the various corners of the maze. Overall, the results indicate that interconnections between the hippocampus and anterior thalamus, via the fornix, help to resolve problems with flexible spatial and temporal cues, but the results also signal the importance of additional, non-fornical contributions to hippocampal-anterior thalamic spatial processing, particularly for problems with more stable spatial solutions.

## Highlights

• Fornix damage mildly impair spatial biconditional and passive place learning tasks.
• Fornix lesions impair spatial go/no-go and alternation problems.
• Fornix lesions impair tests making flexible demands on spatial memory.
• Fornix connections are not always required for learning fixed spatial responses.

## 1 Introduction

Lesions of the hippocampus and the anterior thalamic nuclei produce a similar array of spatial learning deficits in rats. Both structures are, for example, vital for location learning in a Morris water maze, radial-arm maze foraging, and T-maze alternation [4,5,9,16,40,44,47,58,90]. The inference is that these interconnected structures function jointly to enable spatial learning, a view supported by crossed-lesion disconnection studies [32,87,88]. It has, therefore, often been supposed that the hippocampus primarily drives anterior thalamic nuclei activity, principally via its fornical projections (e.g., [1,2,19]). These hippocampal influences essentially comprise the direct fornical projections to the anterior thalamic nuclei, along with the indirect fornical projections via the mammillary bodies [59,70].

The notion that the direct and indirect fornical projections from the hippocampus to the anterior thalamic nuclei are sufficient to explain the importance of their joint interactions for learning and memory can be questioned on several fronts. The first is anatomical. In the rat brain there are some direct projections from the presubiculum and postsubiculum to the anterodorsal and dorsal anteroventral thalamic nuclei that do not involve the fornix [73,74], while the retrosplenial cortex provides an indirect route to and from the anterior thalamic nuclei that again is non-fornical [75–77,82]. The second point relates to the fact that crossed-lesion disconnection studies cannot determine a direction of effect when the two target sites are reciprocally linked. Thus, it is possible that projections from the anterior thalamic nuclei to the hippocampus, which join the cingulum rather than the fornix [21], are critical in regulating their combined role in learning and memory. This notion receives support from recent behavioural studies examining the importance of nonhippocampal inputs to the mammillary bodies for spatial learning [79,80].

Perhaps of more direct concern are the findings from those behavioural studies in which fornix lesions are less disruptive than either hippocampal or anterior thalamic lesions on tests of spatial learning and memory. An influential set of such findings comes from configural learning tasks, which can appear sensitive to hippocampal, but not fornix damage (e.g., [42]). Of particular note are the results from tests of spatial biconditional learning. Although some spatial biconditional problems appear sensitive to hippocampal lesions but not anterior thalamic lesions [61–63], other spatial biconditional tasks are highly sensitive to both anterior thalamic and hippocampal lesions [13,23,66]. Furthermore, the functional link between these two structures for such spatial biconditional tasks has been confirmed by a cross-lesion disconnection study [32]. It is, therefore, striking that fornix lesions can spare those same spatial biconditional tasks that are impaired by both anterior thalamic and hippocampal damage ([24,60,62]; see also [41]). This null result is all the more surprising as fornix lesions will disconnect the hippocampal formation from multiple sites, i.e., not just the anterior thalamic nuclei and mammillary bodies [59].

The start point for the present study was to re-examine the impact of fornix lesions on spatial biconditional learning. The first criterion was to adopt a biconditional task (if in location A choose digging pot X not pot Y, if in location B choose digging pot Y not X) known to be sensitive to both hippocampal and anterior thalamic lesions ([13,23]; see also [32]). The second criterion was to use a task that can be acquired in relatively few trials. A shortcoming with spatial biconditional tasks previously used to address this issue is that they typically require a great many trials before normal rats reach their learning criterion (∼400 trials in [24,41,60]; ∼800 trials in [62]). This feature not only means that task acquisition is incremental and varied, so potentially reducing the ability to detect group differences, but the lengthy training period may increase the likelihood of unintended task solutions by the rats. The present study, therefore, used a location-digging task as in previous studies it had been acquired more rapidly. Furthermore, this task readily lends itself to control comparisons, e.g., learning a matching, nonspatial (contextual) biconditional problem that is spared by both hippocampal and anterior thalamic lesions [13,23], as well as learning location and digging media discriminations. Such tasks were, therefore, used to both cast light on any biconditional learning deficit and to characterise better those spatial tasks that appear to be spared by fornix lesions. The latter goal relates to the issue of how nonfornical pathways may support spatial interactions between the hippocampus and anterior thalamus.

The final experiment extended the study of fornix lesions to another category of spatial task that is sensitive to both anterior thalamic and hippocampal damage [26,37]. This experiment assessed incidental location learning in a water-maze [26,29,35]. The procedure is of particular interest as it forces the rat to navigate according to the spatial disposition of specific maze cues [29,35]. In this task, rats are repeatedly placed on a submerged platform in a square water-maze in which the escape location is signalled by the unique spatial arrangement of cues on the adjoining walls of the pool. Both hippocampal and anterior thalamic lesions disrupt the rat's spatial behaviour on the critical test trial, when the rat is first allowed to swim to find the escape location [26,37]. These findings raise the question of whether fornix lesions would have similar effects. This task appears particularly relevant as previous studies with monkeys indicate that fornix lesions only impair spatial biconditional learning when the subject has to discriminate between scenes that contain common elements [28], an integral feature of this passive learning task in the water-maze [29,35].

## 2 Materials and methods

A total of eight experiments are described in the order in which testing occurred (see Table 1).

### 2.1 Subjects

The study used 27 male Lister Hooded rats (Charles River, Kent, U.K.). At the time of their surgery the rats weighed 277–307 g and were three months old. All rats were housed in pairs under a 12-hour light/dark cycle. The animals were given free access to water, but were maintained at 85% of their free-feeding weight for the duration of the experiments with the exception of the water-maze tasks, where the rats were given food *ad libitum*. All animals were habituated to handling before the start of the first experiment. The experiments were performed in accordance with the UK Animals (Scientific Procedures) Act (1986) and associated guidelines. These procedures were also approved by the appropriate ethics committee at Cardiff University.

### 2.2 Surgical procedures

Rats either received bilateral fornix lesions (Fornix = 15) or sham surgeries (Sham = 12). For the fornix lesions, the rats were first anaesthetised using an isoflurane–oxygen mix. The rat was then placed in a stereotaxic frame (Kopf Instruments, Tujunga, CA), with the incisor bar set at +5.0 mm, and the rat administered with 0.1 mg/kg of the analgesic Metacam (Boehringer Ingelheim Vetmedica, Germany) subcutaneously. A sagittal incision was made in the scalp and the skin retracted to expose the skull. A dorsal craniotomy was made directly above the target region and the dura cut to expose the cortex. The rats received radiofrequency lesions using an OWL Universal RF System URF-3AP lesion maker (Diros Technology Inc., Toronto, Canada). The electrode (0.3 mm tip length, 0.25 mm diameter) was lowered vertically at each site (two in each hemisphere). The electrode tip temperature was then increased to 70 °C for 60 s at lateral sites and 70 °C for 65 s at medial sites. The lesion co-ordinates relative to bregma were anteroposterior 0.0; mediolateral ± 0.7 and ±1.7 from the midline; dorso-ventral −4.5 and −4.6 for the medial site and the lateral sites, respectively.

The control group (Sham) received identical treatments except that the dura was perforated with a 30-gauge Microlance™3 needle (0.3 mm diameter; Becton Dickinson, Drogheda, Ireland) and lowered to +2.0 mm above the target coordinates. Following removal of the probe (fornix lesion) or the needle (sham surgery), the incision was cleaned and sutured. A topical antibiotic powder (Dalacin C, clindamycin hydrochloride, Pharmacia Ltd, Kent, UK) was then applied. All rats also received glucose-saline (5 ml s.c.) for fluid replacement and were then closely monitored in a recovery chamber until they regained consciousness (i.e., movement and righting reflex).

### 2.3 Histological procedure

All rats received a lethal overdose of Euthatal (200 mg/ml sodium pentobarbital, Marial Animal Health Ltd., Harlow, Essex, UK). The rats were initially perfused transcardially with 0.1 M phosphate buffer saline (PBS) followed by a fixative containing 1.5% paraformaldehyde and 1.5% gluteraldehyde in 0.1 M PBS. Brains were postfixed for 4 h at room temperature before being transferred to a cryoprotectant solution containing 25% sucrose in 0.1 M PBS for at least 24 h prior to sectioning. All brain sections were cut in the coronal plane at 40 μm on a sledge microtome. A ‘1-in-4’ series of sections was mounted directly on gelatine-coated slides, and then stained with cresyl violet to allow for histological verification.

### 2.4 Experiment 1: Biconditional discrimination with proximal context cues

**Fig. 1:**
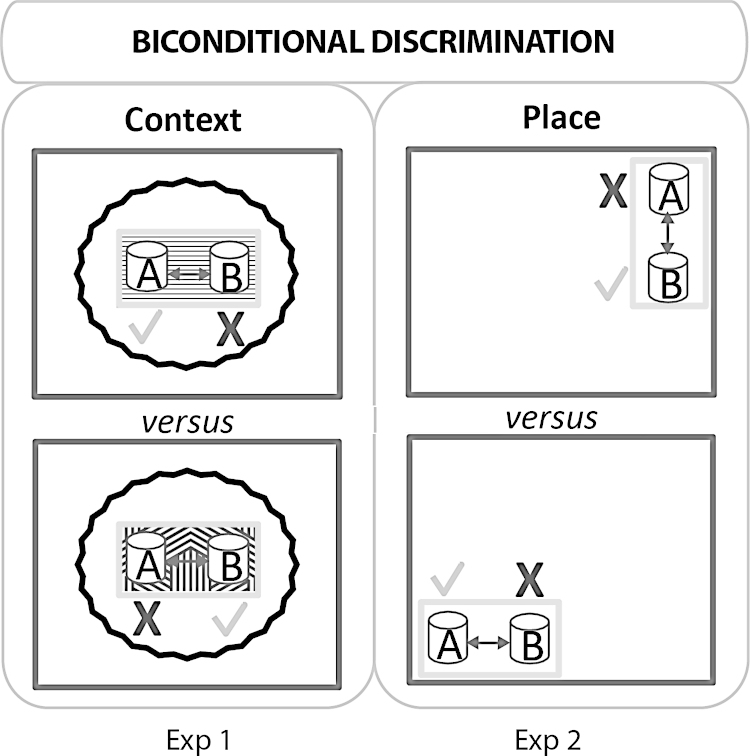
Schematic diagram of the testing protocols for the biconditional discriminations in Experiment 1 (context) and Experiment 2 (place). The dark grey outlines represent the test room, while the smaller grey rectangles represent the plastic test boxes in which the digging cups (A and B) were placed. For the Context Biconditional (Experiment 1) two different test boxes were used, which were placed in the same location with respect to the room. Distal cues were obscured by a curtain (wavy circular line). The correct digging cup (marked by a tick) was determined by the appearance of the test box. The Place Biconditional task (Experiment 2) used just one test box which was placed in one of two locations. The correct digging cup (marked by a tick) was determined by the location of the test box within the room. In both experiments the rat was placed between the two digging cups and, therefore, the rat could approach the digging cups in one of two directions (see arrows). The diagram is not drawn to scale.

The rats were trained on two consecutive biconditional problems. In Experiment 1, local context cues guided the appropriate response, to dig in one of two cups for a food reward (Fig. 1). In Experiment 2, the rats relied on distal spatial cues to guide the correct digging choice (Fig. 1). Only the second problem is impaired by hippocampal and by anterior thalamic lesions [13,23].

#### 2.4.1 Apparatus

Animals were tested in a white, rectangular test box (40 cm long × 20 cm wide × 12.5 cm high) made of plastic. Each digging cup was placed either side of the middle of the short wall of the rectangular box, 22 cm apart (see Fig. 1). The digging cups consisted of a black plastic cylinder with an internal diameter of 7 cm and a height of 6 cm. A grey plastic square (9 cm × 9 cm) was fixed to the base of each cylinder. Velcro secured the cups to the box floor, to discourage the rats from tipping the cups. During pre-training the two cups were identical (both plain black) but, thereafter, the cup containing one medium had a black and white checked outer surface, made by attaching white tape to the outside. The other cup, which contained a different medium, remained plain black. The digging media consisted of either small multi-coloured beads or shredded red paper. The food reward was half of a single Cheerio cereal loop (Nestle, UK), which was buried in one of the digging media. To discourage rats from locating the food reward by its scent, a perforated metal grid was placed inside the cup to create a false bottom. Cereal loops were placed under this grid, where they could not be retrieved by the rats. These loops were replaced with fresh cereals every two days. In addition, cereal crumbs were mixed with the digging medium to ensure that both the correct and incorrect choices smelt of the food reward. Pre-training took place in Room A (330 cm long × 190 cm wide × 256 cm high) where the illumination level was 861 lx

For acquisition of the contextual biconditional task, the rats were tested in a different room (Room B). Room B was square (280 cm long × 280 cm wide × 256 cm high). Throughout training the digging cups were placed in one of two different plastic boxes (both 33 × 26 × 16.5 cm) in the centre of the test room. The two boxes could readily be distinguished as one box had laminated wall panels composed of white and red triangles, and also had a green, textured Duplo (Lego, UK) base covering the floor. The second box had a smooth floor lined with alternating black and white stripes, but with plain walls. The boxes were placed singly on the centre of a table (l02 cm long × 56 cm wide × 76 cm high). An opaque curtain fixed to a circular track on the ceiling was drawn around the test boxes and table to block distal cues. The illumination level in the test boxes was 441.3 lx. The two digging pots with their different digging media were placed 22 cm apart in each box.

#### 2.4.2 Pre-training—Procedure

Rats were placed singly in the white plastic test box with two identical digging cups filled with sawdust in Room A. First a food reward was placed on top of the medium. Then, the reward was buried increasingly deep so that rats had to dig to find the food. Every time the rat found the food the cup was re-baited, and so on for 5–10 min. Both cups were baited. Pre-training lasted between four and six days, when all rats were reliably digging to retrieve the rewards.

#### 2.4.3 Test—Procedure

Three or four rats were simultaneously brought to the test room (Room B) in an enclosed carrying box made of aluminium. Each rat was in a separate container and could not see the surrounding environment. For the biconditional task, a specific digging medium was only correct in a specific context (see Fig. 1). The rats had, therefore, to learn two concurrent rules. Rule 1: multi-coloured beads were correct when presented in the striped floor box (Context 1). Rule 2: red shredded paper was correct when presented in the Duplo base box (Context 2). The combinations of stimuli were counterbalanced across rats. On each trial, a rat was placed midway between the two cups (one to the right, the other to the left) and allowed to choose. A correct choice occurred when a rat dug in the correct cup and retrieved the food. Animals were allowed to put their paws on the medium or to smell the medium before making a choice. An incorrect choice was scored when the rat dug in the unbaited cup, resulting in the removal of the correct cup. The rat was then left for an extra 5 s before being taken out of the box.

Animals received 16 trials per session (8 trials of Rule 1 and 8 trials of Rule 2), with trial types in a semi-randomised sequence such that no more than three consecutive identical trial types could occur, e.g., three trials in Place 1. The rats were run in spaced trials, i.e., groups of three or four rats were run one immediately after the other for every trial. Consequently the inter-trial interval was approximately 4 min. Trial types and the location of the correct cup inside the box (left or right) were counterbalanced within sessions and across groups. The test boxes were always in the same, constant location in Room B, with the surrounding curtain closed. Only the local context changed between trial types. The Sham group performance criterion was set at 80% (for one session), before stopping the experiment. All rats received 14 sessions, one per day.

### 2.5 Experiment 2: Biconditional discrimination with distal spatial cues

#### 2.5.1 Apparatus

The single test box, which contained the digging cups, was identical to the box used during pre-training (white plastic). The task also used the same two cups as Experiment 1 (i.e., one plain black, one black and white checked pattern). Training took place in Room B (same room as Experiment 1), with the test box set on tables (Table 1: 122 cm long × 61 cm wide × 76 cm high; Table 2: 122 cm long × 61 cm wide × 70 cm high) placed in the two diagonally opposite corners of the room, 230 cm apart (Fig. 1). The room was free of obstacles so that all walls were visible from any corner of the room. Posters and shelves were fixed to the walls. The room was illuminated with eight spot bulb lights fixed to the ceiling. The illumination levels in the two corner locations were matched at 215 lx.

#### 2.5.2 Procedure

Using procedures identical to those in Experiment 1, the Fornix and Sham rats were trained on a new biconditional task. Now, the task was to learn which medium was correct in which location. Consequently, the new biconditional rule was that multi-coloured beads (but not shredded paper) were correct in one corner of the room (Place 1), while shredded paper (but not multi-coloured beads) was correct in the diagonally opposite corner of the room (Place 2) (Fig. 1). The single test box was moved between the two locations between trials and its orientation varied in the manner shown in Fig. 1 according to the corner in which it was located. The test box was 16 cm away from the wall when placed on Table 1, and 20 cm away from the wall when placed on Table 2. There was no curtain surrounding the test boxes (unlike Experiment 1), so animals could use distal spatial cues to solve the task. Once again, rats were set down in the middle of the test box at the start of a trial, i.e., placed midway between the two cups (one to the right, the other to the left). Rats were trained for 14 days with 16 trials per day. The training criterion was set at 80% for the Sham group (mean performance over one session). This criterion was reached after nine days. But, in order to verify whether the Fornix animals could learn this critical experiment, training was carried on for five extra days. The total of 14 days matched that required in Experiment 1.

### 2.6 Experiment 3: Spatial go/no-go discrimination

The purpose of this experiment was to determine how readily the rats with fornix lesions could distinguish the room locations used for the biconditional discrimination with distal spatial cues (Experiment 2).

#### 2.6.1 Apparatus

Experiment 3 was conducted in the same room (Room B) and used the same two locations (Place 1; Place 2) as Experiment 2. However for the spatial go/no-go task, only one digging cup was used. The dimensions of the digging cup were identical to those in Experiments 1 and 2, but small white circles (9 mm diameter) were stuck randomly onto the outside of the black digging cup to create a spotted cup. The cup was filled with sawdust and placed in the middle of the shorter wall inside a larger transparent box (52 cm long × 33 cm wide × 17 cm high; Crystal, Whatmore Creative Plastics, 45 LTR, www.whamproducts.co.uk) than those used in Experiments 1 and 2. Both the appearance of the digging cup and the box were changed to reduce transfer effects from the previous experiments.

#### 2.6.2 Procedure

**Fig. 2:**
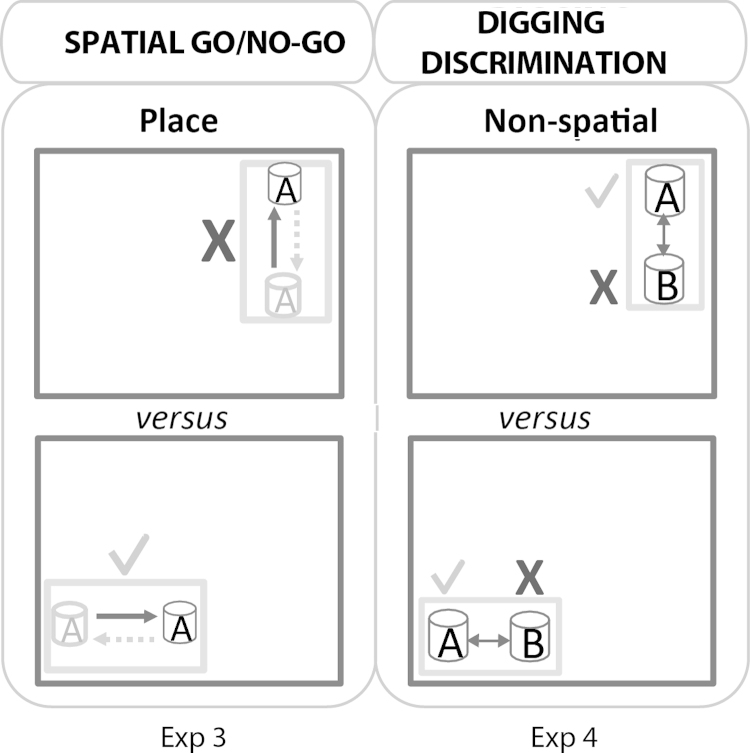
Schematic diagrams depicting the spatial go/no-go (Experiment 3) and non-spatial (Experiment 4) discrimination tasks. The large dark grey outlines represent the test room and the smaller grey rectangles represent the plastic test boxes in which digging cup(s) were placed. Experiment 3: The go/no-go spatial discrimination involved rewarding the rat for digging in one location (tick) but not in a second location (cross). The arrows show the directions the animals ran towards the digging cup. The diagram is not drawn to scale. Experiment 4: The nonspatial discrimination task involved the simultaneous presentation of two different digging media (one always rewarded). The rat was placed in the middle of the test box to start each trial (arrow).

The single digging cup and test box were always placed in one of two table-top locations in Room B. For any given rat, the cup was always baited in one room location (go response), but never baited when placed in the other room location (no-go response; Fig. 2), regardless of the direction the rat approached the cups. As a result, on each trial a single digging cup could be found in four places (Fig. 2): (1) North end of the box in No-go Location, (2) South end of the box in No-go Location, (3) West end of the box in Go Location, and (4) East end of the box Go Location. The rats received 16 trials per day for six days. At the start of each trial, the rat was placed at the end of the box furthest away from the digging cup. Learning was assessed by comparing the latency of the rat to dig when the box was in the baited location and the latency to dig when the box was in the never-baited position. Each trial had a time limit of 20 s, after which the rat was removed. The inter-trial interval was approximately 4 min. If the rat dug in the correct location, the rat was removed as soon as it had consumed the cereal reward, but if the rat dug in the incorrect location it was left for an extra 5 s before being removed from the box. The trial order was counterbalanced pseudo-randomly between the two locations (correct and incorrect; see Experiment 1). In addition, the direction the animal ran to the digging cup (i.e., to the North or to the South end of the box) was also counterbalanced across the 16 trials pseudo-randomly with the following rules: 1) the rat ran to the cup from both directions equally (i.e., 8 trials each), and 2) the rat ran towards the digging cup in the same direction for a maximum of three consecutive trials.

### 2.7 Experiment 4: Discrimination of digging media

This experiment assessed the ability of the rats to discriminate between cups of different appearances containing different digging media. One media/cup combination always contained food (Fig. 2). This ability is a pre-requisite for acquiring the biconditional discrimination.

#### 2.7.1 Apparatus

Experiment 4 was conducted in the same room (Room B) as the previous two experiments. Animals were tested in a white opaque plastic test box (40 cm long × 20 cm wide × 12.5 cm high) that was the same as that used in Experiment 2. The same two (plain black; black and white checked) digging cups as used Experiments 1 and 2 were placed by the shorter walls of the box.

#### 2.7.2 Procedure

For the discrimination task, the black cup contained coloured drinking straws (4.5 cm long) while the cup with the checked pattern contained multi-coloured buttons of varying sizes (5 mm smallest to 11 mm largest). The two cups were presented on each trial, separated by 22 cm. Each trial began by placing the rat in the middle of the test box, equidistant from the two digging cups. Using the same locations as Experiment 2, the rule was that for half of the animals the plastic drinking straws were always correct in both locations 1 and 2, i.e., always baited with food (half a Cheerio loop). For the remaining animals the buttons were correct in both locations (see Fig. 2). Animals received 16 trials per day (eight trials in location 1 and eight in location 2) in a randomised sequence for three days. All other aspects of the procedure matched those used in the preceding experiments, e.g., the inter-trial interval was approximately 4 min.

### 2.8 Experiment 5: Place/Direction learning in a cross-maze

**Fig. 3:**
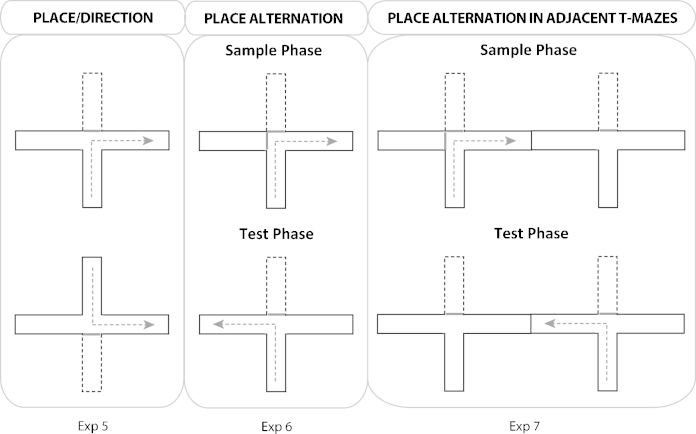
Schematic diagrams of the test protocols for the Place/Direction discrimination (Experiment 5), reinforced Place Alternation (Experiment 6), and reinforced Place Alternation in adjacent T-mazes (Experiment 7). All three experiments used a cross-maze that could be converted to a T-maze by the addition of a barrier (grey bar). For the Place/Direction discrimination (Experiment 5) rats were rewarded in a constant location that was in a constant direction from the choice point. The upper and lower figures show two test configurations while the arrows depict the correct choices. For the reinforced Place Alternation (Experiment 6) each trial consisted of a sample phase followed by test phase. The reinforced rule was to select in the test phase the arm opposite to that entered in the sample phase (see arrows). For reinforced Place Alternation in adjacent T-mazes (Experiment 7) the test protocol was the same as for Experiment 6 except that the sample and test phases were in adjacent mazes. For half of the trials the correct choice in the test phase brought the animal back into the same region as in the sample phase (a ‘same’ trial, as depicted in Fig. 3). The remaining trials were ‘different’ trials.

The next three experiments examined different aspects of spatial learning in a cross-maze. In Experiment 5, rats were rewarded for turning into the arm that remained in a constant location in the room (e.g., East; Fig. 3), so testing spatial reference memory. As the rats started each trial from either North or South it meant that the correct location was in a constant direction from the choice point (e.g., East) but varied as to whether it involved a left or a right turn (i.e., it differed egocentrically). After completing the previous experiment, one Fornix rat became ill and was perfused because of health concerns. Consequently, for Experiments 5–8 there were 14 rats in the fornix lesion group.

#### 2.8.1 Apparatus

Pre-training and testing took place in a room (300 cm × 280 cm × 240 cm; Room C) different from those used in the previous experiments. The room contained a variety of extra-maze cues (e.g., posters, tables, door) and was illuminated by two fluorescent strip lights. The apparatus consisted of a four-arm (cross-shaped) maze. The walls of each arm (70 cm long × 10 cm wide × 17 cm height) were made of clear Perspex. The floor of the arms was made from wood and painted white. A sunken food well was located at the end of each arm. An aluminium barrier could be positioned 25 cm from the end of each arm to create a start area. The maze was supported on two metal frames (94 cm height) and was situated in the middle of the room.

#### 2.8.2 Pre-training

During both pre-training and testing, the rats were transported inside a light-tight aluminium carrying box where they also remained between trials. During pre-training, the cross-maze was blocked at the central junction with a metal barrier, creating three straight alleys: 1) a start arm alley (South arm), 2) both the choice arms (i.e., the top of the “T”; East and West arms) and 3) the North arm (opposite the start). Rats were trained to eat in these straight alleys, ensuring that they were not rewarded for specific arm turns by the placement of the metal barriers. On the first day of pre-training, rats were placed in pairs in the maze for 10 min, on day two of training and, thereafter, each rat was placed for five minutes in an alley with sucrose pellets (45 mg per pellet; Noyes Purified Rodent Diet, Lancaster, NH, USA), initially scattered along the floor, but later placed within the food wells. Pre-training continued until the rats readily walked down the alley to eat the pellets in the food well.

#### 2.8.3 Testing

The rats, which received twelve trials per day for eight days, were rewarded for selecting the arm in a constant location (Fig. 3). The rat was deemed to have made a choice when it placed a hind foot down an arm. Following a correct choice, the rat was allowed to eat the reward before being returned to the metal carrying case. When the rat made an incorrect choice it was allowed to run down the entire length of the incorrect arm to reach the empty food well, before being returned to its carrying case. The rats were run in squads of 3–4, each rat receiving one trial at a time. Consequently, the inter-trial interval was approximately 4 min. The maze was rotated after each trial in a random sequence of 90°, 180° and 270° to remove intra-maze cues.

The South start arm and North start arm were each used six times per session in a random sequence. For half of the cohort, the rule was when released from the South, turn left (i.e., West) and when released from the North, turn right (i.e., West). For the remaining rats the opposite rule was reinforced, i.e., always go East. On session one only, all three arms were open for the rat to select. Thereafter, the rats could only select either the East or the West arm. The change in procedure reflected concerns that the two groups might be differently biased to just run straight.

### 2.9 Experiment 6: Place alternation in a T-maze

The task, reinforced place alternation, provides a test of working memory. This task was selected as it has repeatedly been shown to be sensitive to fornix damage (e.g., [8,12,41,54]), so helping to confirm the effectiveness of the present surgeries and the stability of any deficit.

#### 2.9.1 Apparatus

Pre-training and testing took place in a different room (304 cm × 290 cm × 239 cm; Room D) from those used in the previous experiments. The room contained a variety of extra-maze cues (e.g., posters, tables, door) and was illuminated by two fluorescent strip lights (140.8 lx in the centre of the room).

Two identical cross-mazes, which could be modified to form a T-maze, were used. The walls of each of the four arms of the two mazes (45.5 cm long × 12.0 cm wide × 32.5 cm high) were made of black Perspex. (The mazes had opaque walls to reduce confusion when two mazes were used side by side—see Experiment 7.) The floors of the two mazes were made of wood and painted white. A sunken food well (2 cm in diameter and 0.75 cm deep) was located at the end of each arm. By placing an aluminium barrier at the entrance of an arm it was possible to prevent access to that arm. The mazes were placed on a table 74 cm high. During pre-training, which lasted two days, the mazes were placed side by side so that the East arm of the left maze (Maze A) touched the West arm of the right maze (Maze B).

#### 2.9.2 Procedure

The rats were given eight trials per day for four days. Each day, four trials were given in one T-maze while the other four trials used the second T-maze. The two mazes remained side by side on the table so that the East arm of Maze A just touched the West arm of Maze B. Each trial consisted of a sample phase and a choice phase in the same maze. During each sample phase, the rat was only allowed to enter one of the arms at the top of the “T” by blocking the entrance to the opposite arm at the central junction in the maze. The rat could then consume the single sucrose pellet (45 mg) in the food well at the end of the sample arm. The rat was then picked up and confined in the same start arm for approximately 15 s (Fig. 3) while the barrier at the choice point was removed. The rat was then allowed to run to the choice point, where it had free access to the two arms of the T-maze. The rat was rewarded with a single 45 mg sucrose pellet for choosing the arm not previously visited during the sample phase, i.e., the rat alternated arms between the sample and choice runs (Fig. 3). All other procedural details, e.g., spaced trials, were identical to Experiment 5.

### 2.10 Experiment 7: Place alternation in adjacent T-mazes

The task and test room were identical to those for Experiment 6, apart from one key difference. Now the sample and test phases occurred in separate mazes, which were placed side-by-side (Fig. 3). Consequently, the procedure should remove intra-maze cues that might guide choice behaviour (see [52]).

#### 2.10.1 Apparatus

The mazes and their positioning were identical to that for Experiment 6.

#### 2.10.2 Procedure

As in Experiment 6, each trial had a sample phase and a choice phase. However, the rats now received the sample phase in one maze (e.g., Maze A) and the choice phase in the other maze (e.g., Maze B). This procedure was randomised so that the sample phase could be in either Maze A or Maze B. During the choice phase, the rat was rewarded for going into the arm that involved the opposite direction of travel to that in the sample phase, which is also the direction that faces the opposite wall and involves the opposite body turn to that in the sample phase, e.g., left then right (see Fig. 3). The rats received eight trials per day for eight days, with an inter-trial interval of approximately 4 min.

### 2.11 Experiment 8: Passive place learning in a cue-controlled swim-maze

The final experiment involved learning the location of an escape platform that was immediately below the water surface, i.e., not visible. Unlike most water maze studies, the rats learnt the location information passively. That is, they were repeatedly placed on the escape platform but not allowed to swim in the pool to that location until the first test probe. The rats were, however, initially pre-trained to find a submerged platform in a circular pool in one test room, before being tested in the square pool (passive learning) in a second room (see Table 1). Pre-training was required in order to ensure that all rats would search for an escape platform during the critical test probes (see [17]).

#### 2.11.1 Pre-training

Pre-training took place in a 2 m diameter watermaze in a room different to that used the actual experiment (Table 1; 360 cm × 300 cm × 240 cm; Room E). All rats received four pre-training sessions in the circular pool with curtains closed to block room cues. For these pre-training sessions the platform was placed in a quadrant (W, E, S, N, NE, NW, SW, or SE), with each location used twice throughout the four sessions, but not within the same session. For each session the rats were carried into a room adjacent to the pre-training room in groups either three or four in a light-tight aluminium carrying box. The rats remained in this box between trials.

For pre-training, the platform was randomly positioned either 20 cm or 40 cm from the edge of the pool, each for two trials per session. The rats were also randomly released from a start position (W, E, S, N, NE, NW, SW, or SE), with each location used twice throughout the four sessions, but not within the same session. The rats were required to swim to the platform. For the first two sessions, a beacon was attached to the escape platform. For Session 3 and 4, the platform had no beacon, and no cues were available to the rat. The rats had a maximum of 120 s to find the platform on the first three sessions, and 90 s during Session 4. If the rats successfully found the platform, they remained on the platform for 30 s before being returned to the carrying box in the adjacent room. However, if the rats did not find the platform, the experimenter showed the rat the location of the platform by tapping gently on it, in the first instance, or guiding the rat (they would follow the experimenter's hand through the water) to the platform where the rat remained for 30 s. The rats completed one session of four pre-training trials each day.

#### 2.11.2 Apparatus and room for training and test trials

**Fig. 4:**
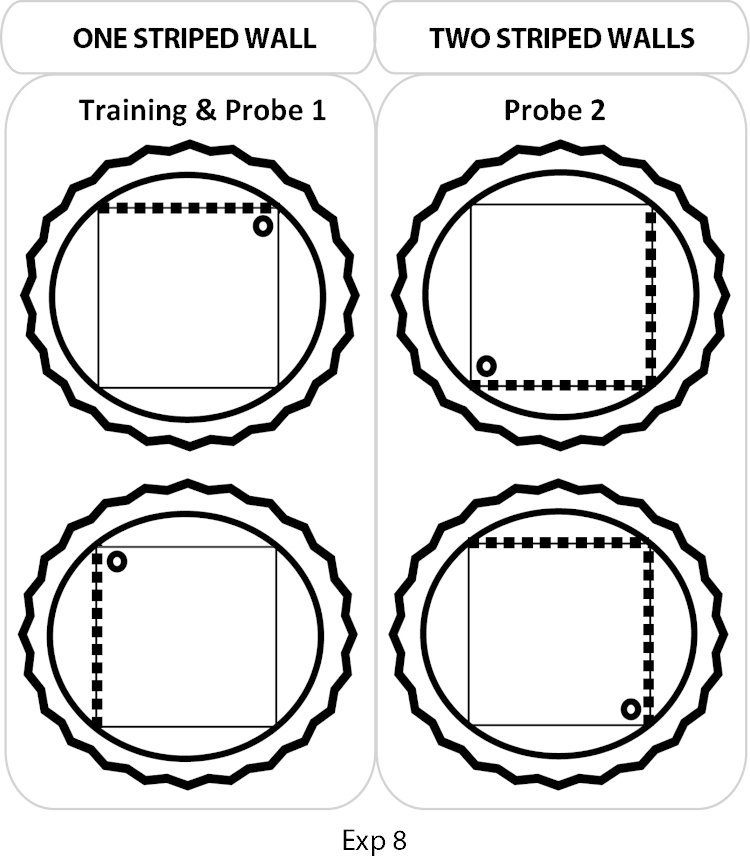
Schematic diagram of the passive place learning task in a swimming pool (Experiment 8). The inner shape depicts the square pool, the surrounding circle is the larger pool within which the smaller pool is placed, and the rippled circle represents the curtains used to block distal cues. Each inner pool was rotated on consecutive trials, as indicated in the figure. For the square pool the thick dashed lines represent the striped walls. During training and for the one striped wall probe there was only one striped wall. For the two striped wall probe, two striped walls were placed side by side. The small circle represents the platform on which the rat would be placed passively during the training trials. The platform was not, however, present during either Probe 1 or Probe 2. Consequently, the circles represent the ‘correct’ corner in the swim pool.

The experiment used a square pool set within a larger white, circular swim pool, measuring 2.0 m in diameter and 60 cm deep (Fig. 4). The pool stood 60 cm above the floor in the centre of the test room (430 cm × 400 cm × 240 cm; Room F). The pool was filled with water to a depth of 30 cm and was maintained at a temperature of 24 °C (±2 °C). The water was made opaque by adding 0.5 l of white opacifier (Opulyn 303B, Dow, USA; Cat No. 10318500), which was changed daily.

Throughout the experiment, rats were trained in a square-shaped pool constructed of three white Perspex boards (140 cm long, 50 cm high, and 2 mm thick) and one black and white striped Perspex board (140 cm long, 53 cm high, and 2 mm thick). The vertical black stripes were 10 cm wide with 10 cm white intervals between stripes. The black stripes began 5 cm from the side edge of the board. Each board was placed vertically in the pool and suspended by bars that extended over the edge of the pool to create the square-shaped pool. The test configuration (Fig. 4) created three sets of corners: (1) black and white striped wall to the left of the white wall, (2) black and white striped wall to the right of the white wall, and (3) white wall meeting white wall (two of these corners; see Fig. 4).

A white circular false ceiling (2.0 m in diameter) was suspended 1.6 m above the floor of the pool. A video camera fixed to the centre of the ceiling recorded the rats’ movements, which were analysed using Watermaze software [48]. Eight, 45-W lights (22.5 cm in diameter) located in the circular ceiling illuminated the pool. The lights were equidistant from each other in a 160 cm diameter circle, whose centre was the same as the centre of the circular ceiling. An escape platform (10 cm in diameter) was mounted on a column, which resulted in the platform being submerged 2 cm below the water surface. A white curtain, which was attached to the edge of the circular ceiling, was drawn completely around the pool during all training and test trials, so hiding distal room cues. The curtain was 150 cm high and fell 25 cm below the edge of the pool. There was a door (166 cm by 205 cm) in the centre of the South wall connecting the swim pool room to the control room, which contained the computer equipment used to monitor the rats’ behaviour.

#### 2.11.3 Training and first probe–One striped wall

Following pre-training, the rats were passively trained for eight days, each with four training trials, in the square pool. The pool platform was positioned 25 cm from a corner on an imaginary line that bisected the corner. The position of the platform was counterbalanced, so that half of the rats from each group had the platform placed in a corner where the striped wall was to the right of a white wall and the other half experienced the platform in the corner where the striped wall was to the left of the white wall (see Fig. 4). (The platform was never located at a white-white wall corner.) Between each trial, the square pool was randomly rotated 90°, 180°, or 270° clockwise. Four possible orientations were used (North, South, East or West) with each orientation being used once for any given session.

For each session, the rats were carried into a room adjacent to the test room in groups of either three or four in a light-tight aluminium carrying box. The rats remained in this box between trials. Each rat was placed individually on the escape platform, where it remained for 30 s undisturbed, before being removed, dried and returned to the holding box.

On the eighth day (final session), the rats received three training trials followed by a probe (Test trial ‘One striped wall’), where the platform was removed and the animal was allowed to swim for 60 s in the square pool (Fig. 4).

#### 2.11.4 Training and transfer probe—Two striped walls

The rats were passively trained for an additional day as described above with one striped wall, before the next probe (Test trial ‘Two striped walls’) that now used two black and white striped walls arranged next to each other for the first time (Fig. 4). The first three trials involved standard passive training with the one striped wall, but this was replaced by the two striped condition on the fourth trial when the rat was put into the water in the centre of the pool and allowed to swim for 60 s in the absence of the platform. This new configuration created four different corners: (1) black and white striped wall to the left of the white wall, (2) black and white striped wall to the right of the white wall, (3) the junction of two striped walls, and (4) the junction of two white walls. In all other respects, the probe test was identical to the one striped wall condition.

#### 2.11.5 Statistical analysis

The first corner each rat approached for each of the probe test was recorded (i.e., correct or incorrect corners) as well as the latency to the correct corner. Circular search zones in each of the four corners were then used to analyse further the results from the test trial [48]. Each zone had a diameter of 30 cm with its centre positioned 25 cm from a corner on a line that bisected the corner, i.e., where the centre of the platform would have been located. The time spent in the correct zones (i.e., the corner where the platform was located during training) was compared with the remaining corners using an ANOVA with one between-subject factor (Group) by one within-subject factor (Corner: correct; incorrect). The times spent in each corner were treated as independent as an appreciable amount of time was also spent in the remainder of the pool.

For the one striped wall probe, only the correct and incorrect stripe-white corners were compared to assess whether fornix lesions disrupt learning their structural configuration (i.e., stripe-white vs. white-stripe). The remaining two incorrect white-white walls were excluded from the analyses as they were rarely visited. For the subsequent two striped walls test probe, all four corners were first analysed. Next, just the two mirror-imaged corners (stripe-white vs. white-stripe) were analysed, so giving a direct comparison with the one striped wall test. The mean swim speed (cm/s) and the mean distance travelled (cm) were also examined (two-tailed). Fisher's Exact Probability was used to compare whether the two groups differed from one another in their first corner choice (correct or incorrect).

## 3 Results

### 3.1 Histological findings

**Fig. 5:**
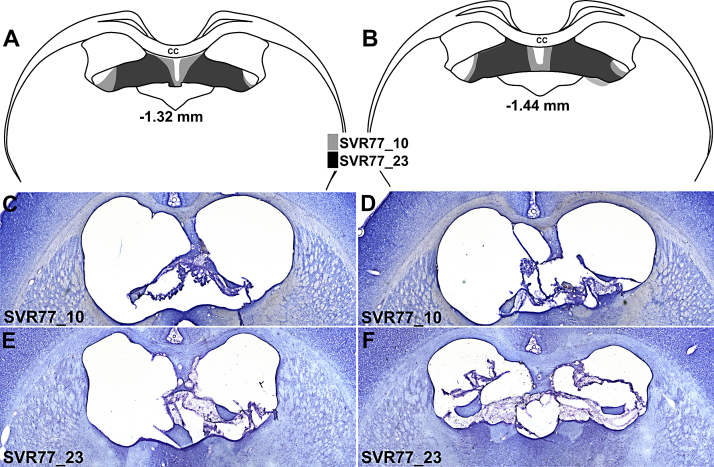
Two coronal sections that depict those cases with the smallest (dark grey, ‘SVR77_23′) and the largest (light grey, ‘SVR77_10′) extent of fornix damage. (A) and (B) provide schematic depictions of the lesion extent. The numbers refer to the approximate distance of the sections in mm caudal to bregma. Abbreviations: cc, corpus callosum. (C) and (D) are photomicrographs (Nissl stain) of coronal sections taken from the case with the largest fornix lesions. (E) and (F) are photomicrographs (Nissl stain) of coronal sections taken from the case with the smallest fornix lesions.

The fornix lesions were consistently centred in the tract just before it descends into the septum (Fig. 5). The lesion damage was very restricted in that did not involve the corpus callosum or any cortex, aside from probe tracks. In five rats there was appreciable sparing to the fornix in at least one hemisphere and so these cases were removed from the statistical analyses, leaving ten cases in the Fornix lesion group. The lesions in the largest and smallest of the remaining ten cases are depicted in Fig. 5. The tissue damage in these cases was typically confined to the fornix with only unilateral damage being found in the most dorsal part of the anteroventral thalamic nucleus (one case), anterodorsal thalamic nucleus (one case), and stria medullaris (one case). All 10 cases suffered extensive damage to the fornix, though there was a narrow band of spared tissue under the midline of the corpus callosum, which varied in extent. Any other sparing occurred at the most lateral tip of the fornix/fimbria, which was always highly distorted. Even in the case with the largest lesion, there was a fragment of tissue at the extreme lateral portion of the tract (Fig. 5). The median amount of sparing at this level involved approximately 15% of the entire tract (coronal plane).

### 3.2 Experiment 1: Biconditional discrimination with proximal context cues

**Fig. 6:**
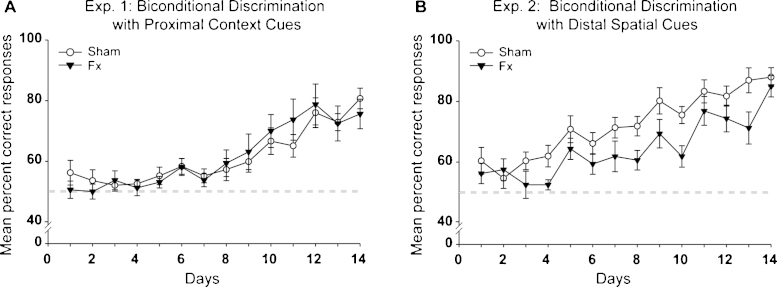
Biconditional discrimination performance. The mean percent correct responses of the fornix lesion (Fx) and control (Sham) rats over 14 successive test sessions. (A) Performance on the Context Biconditional discrimination (Experiment 1). (B) Performance on the Place Biconditional discrimination (Experiment 2). The light grey dashed line depicts chance (50%). Both graphs show the mean scores ± their standard error.

The fornix lesions did not appear to disrupt the acquisition of a biconditional association between a particular digging medium (cup) and the contextual cues provided by different test boxes (Fig. 6 left). The Fornix group did not differ from the Sham controls (*F* < 1), both showing clear task acquisition over the test days (*F*_(13, 260)_ = 16.23, *p* < 0.001). The group by test day interaction was not significant (*F* < 1).

### 3.3 Experiment 2: Biconditional discrimination with distal spatial cues

The fornix lesions lowered overall performance on this task (*F*_(1, 20)_ = 4.52, *p* = 0.046) but did not stop the rats from learning the biconditional associations (Fig. 6 right). Consequently, performance improved across days (*F*_(13, 260)_ = 23.38, *p* < 0.001) and there was no interaction between group performance and test day (*F*_(13, 260)_ = 1.27, *p* > 0.1). Both the Sham and Fornix groups were above the 80% criterion by the final test day and did not differ on this final test day (*F* < 1).

The final five test days from both the proximal context cues biconditional task (Exp. 1) and the distal spatial cues biconditional task (Exp. 2) were compared using a two within-subjects factors (test day; context or spatial biconditional) and a between subject factor (group) ANOVA. These analyses yielded a significant Group × Condition (spatial, context) interaction (*F*_(1, 20)_ = 9.81, *p* = 0.005). Examination of the simple effects indicated that the Sham group performed significantly better on the distal spatial cues biconditional task compared with context biconditional (*F*_(1,20)_ = 18.75, *p* < 0.001), whereas the Fornix group performed both tasks equally (*F* < 1). The same analysis revealed a main effect of condition (*F*_(1, 20)_ = 8.95, *p* = 0.007), as the place biconditional was solved more readily, but no main effect of group (*F* < 1). When considering these tasks effects it should be noted that the order of testing was not counterbalanced, i.e., all rats received the context problem first.

### 3.4 Experiment 3: Spatial go/no-go discrimination

**Fig. 7:**
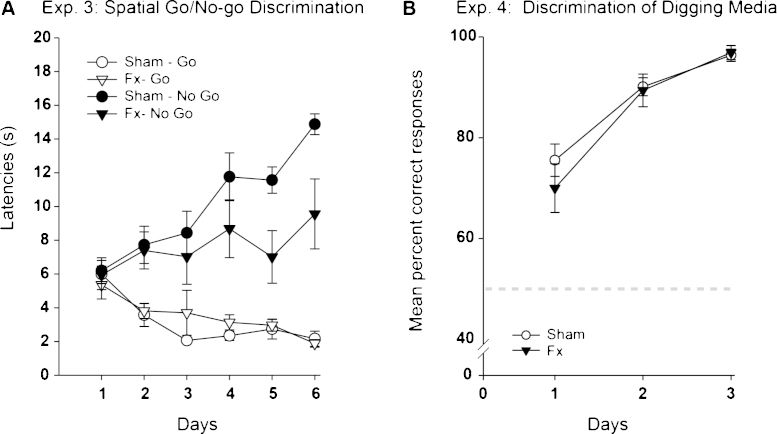
(A) Performance on the spatial go/no-go discrimination (Experiment 3). The graph shows the mean latencies (s) during go and no-go trials of the rats with fornix lesions (Fx) and the control (Sham) rats. (B) Digging media discrimination (Experiment 4). The graph shows the mean percent correct responses for each of the three test days. The light grey dashed line depicts chance (50%). Both graphs show the mean scores ± their standard error.

Fornix lesions disrupted performance in an asymmetric manner. While the latency scores to dig on the ‘go’ trials appeared unaffected, the Fornix rats responded prematurely on the ‘no-go’ trials (Fig. 7). These data were examined using a mixed model ANOVA with the between subjects factor Group (Fornix, Sham) and the within subject factors Condition (Go, No-go) and Days.

The mixed model ANOVA yielded a significant Group × Condition (go/no-go) × Days interaction (*F*_(5, 100)_ = 2.76, *p* = 0.039, Greenhouse-Geisser correction), reflecting the emergence of different patterns of performance between the two groups. There were also significant Condition × Day (*F*_(5, 100)_ = 24.58, *p* < 0.001) and Group × Condition (*F*_(1, 20)_ = 4.84, *p* = 0.04) interactions. The latter interaction showed how the deficit in the Fornix group was largely confined to the no-go trials, i.e., the lesions affected the ability of the rats to withhold responding, but the groups did not differ significantly during the go trials. The main effect of Condition (go vs. no-go) was significant (*F*_(1_, _20)_ = 74.5, *p* < 0.001), reflecting the ability of the rats to discriminate the test locations. All other main effects and interactions failed to reach significance (though main effect of day: *p* = 0.06, Greenhouse Geisser correction; rest: *p* > 0.1).

The experimental design meant that for half of the trials, the rats’ direction of movement pointed them towards the ‘same’ corner within the test room (see Fig. 2). In the remaining trials the rats headed towards ‘opposite’ corners. When performance (ratio of time for no-go divided by go trials) was separated into ‘same’ and ‘opposite’ trials for the last two sessions it was found that the Fornix group were impaired on both trial types (both *p* ≤ 0.026) and there was no interaction between lesion and trial type (*F* < 1).

### 3.5 Experiment 4: Discrimination of digging media

Both groups rapidly acquired this simultaneous discrimination over the three days of testing (Fig. 7), with no evidence of a fornix lesion effect (*F* < 1). By the second day the mean percent correct scores for both groups were above 80%, which increased to over 90% on the final day.

### 3.6 Experiment 5: Place/Direction learning in a cross-maze

**Fig. 8:**
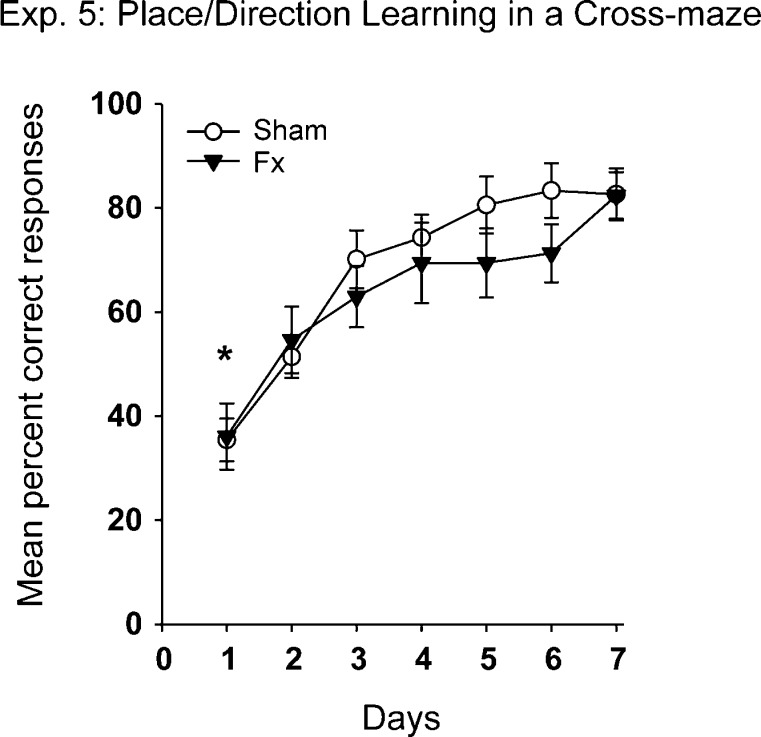
Performance on the Place/Direction learning discrimination in a cross-maze (Experiment 5). The graph shows the mean percent correct responses (±standard error) of the rats with fornix lesions (Fx) and their controls (Sham). On day 1, chance performance was 33% (marked by asterisk) but for all following days it was 50%.

Both groups learnt this spatial task at a comparable rate. The mean percent correct responses across test days for the Fornix and Sham rats (Fig. 8) show how both groups improved across test days (*F*_(7, 133)_ = 23.32, *p* < 0.001), with no overall group difference (*F* < 1) and no Group × Day interaction (*F* < 1).

### 3.7 Experiment 6: Place alternation in a T-maze

**Fig. 9:**
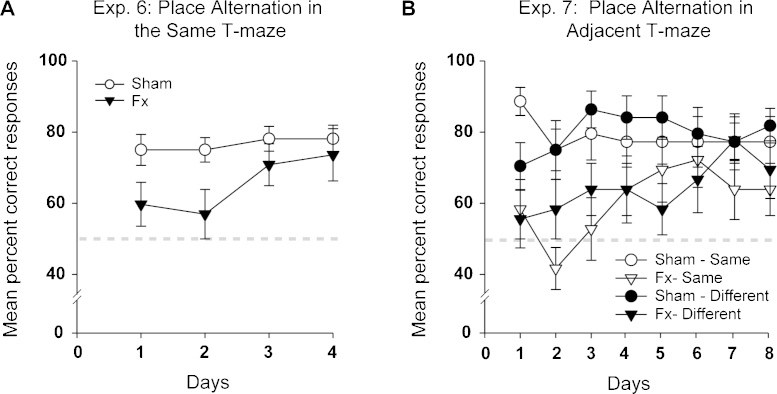
Mean group performance for reinforced Place Alternation (Experiment 6) and reinforced Place Alternation in adjacent T-mazes (Experiment 7). The graphs shows the mean percent correct responses (±standard error) of the rats with fornix lesions (Fx) and their controls (Sham). Chance performance was 50%. For Experiment 7 the results are divided into ‘same’ and ‘different’ trials. On ‘same’ trials the correct arm choice brought the rat close to the sample location (see Fig. 3 right).

The Fornix rats were significantly impaired when compared with the Sham group (*F*_(1, 19)_ = 6.93, *p* = 0.016). On Day 1 the Sham rats performed around 75% reflecting how the task immediately benefited from the spontaneous alternation bias, while the Fornix rats only performed around 60% correct (Fig. 9). Over the four test sessions there was no main effect of Day (*F*_(3, 57)_ = 2.23, *p* = 0.094) and no Group × Day interaction (*F* < 1). The scores of the Fornix group did not differ from those of the Sham rats on test days 3 and 4 (simple effects, smallest *p* = 0.32).

An additional comparison grouped the individual performance of each rat by trial position, so that the scores on trials 1–8 could be compared. There was a main effect of trial number (*F*_(7, 133)_ = 3.79, *p* = 0.001) reflecting poorer overall performance on some of the trials. There was also a group by trial position interaction (*F*_(1, 33)_ = 2.34, *p* = 0.028) as the rats with fornix lesions appeared more affected by trial number within a session (most evident for Trial 6). The scores of the Fornix rats remained lower than those of the Sham rats on trial one (the trial with the least proactive interference), although this difference was not significant (simple effects *F*_(1, 152)_ = 3.00, *p* = 0.085).

### 3.8 Experiment 7: Place alternation in adjacent T-mazes

This procedure removes intra-maze cues that can be used to guide performance. The trial types can be divided into those where the correct choice required running towards the same general location in the middle of the test room for both the sample and test phases (e.g., sample Maze A, East arm and test Maze B, West arm) or running to towards opposite sides of the room (e.g., sample Maze A, West arm and test Maze B, East arm). The two trial types are called “Same” or “Different,” respectively (see Fig. 3 for an example of a ‘Same’ trial).

Like the previous experiment, the Fornix rats were impaired on spatial alternation (main effect of Group, *F*_(1, 18)_ = 13.24, *p* = 0.002; Fig. 9). No other main effects or interactions were significant (all, *p* > 0.1) in the mixed model ANOVA with the between subjects factor Group (Fornix, Sham) and the within subject factors Condition (Same, Different). Consequently the two trial types did not differ in overall difficulty, nor were the Fornix rats particularly impaired by one of these two trial types.

### 3.9 Experiment 8: Passive place learning in a cue-controlled swim-maze

#### 3.9.1 Training and first probe—One striped wall

The numbers of rats first selecting the correct corner were 10/12 (Sham) and 8/10 (Fornix), and so this measure did not distinguish the groups. Likewise, the latency to first reach the correct corner failed to show a group difference (*t*_(20)_ = 1.34, *p* = 0.20, two-tailed). This second comparison was complicated by the finding that the Fornix rats swam at a higher speed (*t*_(20)_ = 3.07, *p* = 0.006) during the probe test, but analyses based on distance travelled to first reach the correct corner also found no evidence of a lesion effect (*t*_(20)_ = 1.01).

**Fig. 10:**
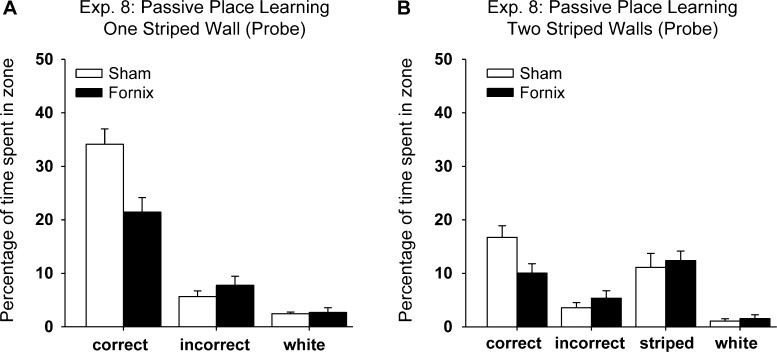
Passive place learning in a square pool (Experiment 8). (A) Performance on the first probe test (‘one striped wall’) was given after passive training with one striped wall and three white walls (see Fig. 4). The histogram show the percent of all swim time spent in each of the three types of corner. (The data for the two white corners has been combined). The ‘correct’ corner corresponds to where the escape platform had been located during training while the ‘incorrect’ corner was its mirror-image. (B) Performance on the second probe trial (‘two striped walls’) after one more session of passive training (see Fig. 4). The histogram shows the percent of all swim time spent in each corner. The pool now had two striped walls and two white walls. Consequently there were four distinct corners: (1) the ‘correct’ corner corresponds to where the escape platform had been located during training, (2) the ‘incorrect’ corner was its mirror-image, (3) the ‘striped’ corner was the novel corner formed by the meeting of two striped walls, and (4) the ‘white’ corner, where two white walls met. Data shown are group means, while the vertical bars are the standard error of the means.

Fornix lesion effects were, however, more apparent when the entire probe trial was considered. The mean percentages of time spent in the correct and incorrect corners for the Fornix and Sham groups on the first Test Trial (Fig. 10, One striped wall) revealed a significant Group × Corner (Correct, Incorrect) interaction (*F*_(1, 20)_ = 8.15, *p* = 0.01). The Sham group spent significantly more time in the Correct corner compared with the Fornix group (simple effects *F*_(1, 40)_ = 16.15, *p* < 0.001), although the two groups did not differ in the amount of time spent in the Incorrect corner (*F* < 1). Both groups also spent significantly more time in the Correct compared with Incorrect corners (Sham: *F*_(1, 20)_ = 60.6, *p* < 0.001; Fornix: *F*_(1, 20)_ = 14.04, *p* = 0.001), suggesting that while the Fornix group could discriminate between these mirror-imaged corners, they did not show as strong a preference for the Correct corner as the Sham rats. The main effect of Group and the main effect of Corner were also significant (Group: *F*_(1, 20)_ = 8.60, *p* = 0.008; Corner: *F*_(1, 20)_ = 66.5, *p* < 0.001). The former effect reflects how the Sham rats spent more time in the corners of the maze. The final comparison showed that the Fornix rats swam a greater distance than the Sham rats during the probe test (*t*_(20)_ = 3.11, *p* = 0.006).

#### 3.9.2 Training and transfer probe—Two striped walls

The numbers of rats first selecting the correct corner were 4/12 (Sham) and 2/10 (Fornix), proportions that did not distinguish the groups. These proportions were appreciably lower than those from the first probe (One striped wall) because of the attraction shown to the novel striped–striped corner (Fig. 10). Comparisons based on the latency to first swim to the correct corner also failed to show a group difference (*t* < 1). Additional comparisons showed that across the probe trial there was no group difference for either swim speed or total distance travelled (both *t* < 1).

For the entire probe trial, the total times spent in the four corners (Correct, Incorrect, Stripe-Stripe, White-White; Fig. 10) did not distinguish the two groups (*F* < 1) and there was no Group by Corner interaction (*F*_(3, 60)_ = 2.44, *p* = 0.11, Greenhouse Geisser correction). The main effect of Corner was significant (*F*_(3, 60)_ = 20.9, *p* < 0.001). However, as for the first probe, lesion effects became apparent when just the mirror-imaged corners where considered. When just the Correct and Incorrect corners, i.e., the two mirror-imaged corners, are compared there is a significant Group by Corner interaction (*F*_(1, 20)_ = 6.03, *p* = 0.023), but no group difference (*F*_(1, 20)_ = 2.36, *p* = 0.14) as the overall times spent in these two corners did not distinguish the groups. The simple effects showed that the Sham group spent more time in the Correct corner than the Fornix group (*F*_(1, 40)_ = 8.10, *p* = 0.007), though there was no group difference for the Incorrect corner (*F*_(1, 40)_ = 0.57, *p* = 0.45).

## 4 Discussion

The present study examined the impact of fornix damage in rats with reference to what is known about the effects of lesions in the hippocampus and anterior thalamic nuclei on spatial learning. While the fornix interconnects the hippocampal formation with numerous structures [59] it has been argued that the direct hippocampal projections to the anterior thalamic nuclei, along with the indirect fornical projections via the mammillary bodies, are of especial importance for spatial memory in rodents and episodic memory in humans [1,3,19]. One pertinent issue concerns the impact of fornix lesions on spatial biconditional problems, as such tasks are often highly sensitive to both anterior thalamic and hippocampal damage (e.g., [13,23,66]), including when tested in a crossed-lesion disconnection study [32], yet appear to be spared after fornix lesions [24,41,60,62]. This task formed the start point for a series of experiments that first re-examined the impact of fornix lesions on spatial biconditional learning, using a task that might address some of the potential shortcomings in previous studies. This task was followed by a series of other spatial studies that sought to characterise those spatial processes which might involve nonfornical links between the hippocampus and anterior thalamic nuclei.

The first two experiments showed that the fornix lesions spared acquisition of a contextual biconditional problem but produced a mild deficit on the spatial biconditional problem (if in place A dig in pot X, if in place B dig in pot Y). Both tasks were mastered by the control rats in about 200 trials, an appreciably faster rate than that reported in previous, corresponding studies [24,41,60,62]. While this contrasting pattern of spared (contextual) and impaired (spatial) learning in the present study seemingly echoes the effects of anterior thalamic lesions and hippocampal lesions on the corresponding tasks [13,23], there is a marked difference. The impact of fornix lesions on the spatial biconditional task appeared appreciably less severe than that seen after these other limbic lesions, with clear evidence of task acquisition despite the fornix surgery (Fig. 6). In contrast, rats with either anterior thalamic lesions [23] or hippocampal lesions [13] remained close to chance when transferred from the contextual to the spatial biconditional problem (see also [60,66,67]). Minor procedural differences between these various studies preclude direct quantitative comparisons, yet all of the evidence points to a much milder deficit after fornix lesions than that seen after either anterior thalamic or hippocampal lesions [13,23]. One sign of this relative sparing is that the performances of the Fornix and Sham groups did not differ on the final day of spatial biconditional training (Fig. 6).

The present study also explored various elements of the spatial biconditional problem. The normal acquisition of the contextual biconditional task (Experiment 1) revealed that the fornix lesions did not alter the ability to use local cues to guide differential responses. In this contextual biconditional task the rat only needed to know whether a given contextual cue (e.g., a floor surface) was present or absent, i.e., no added spatial/geometric dimension was required (see also [13,23,28,33,49]). The fornix lesioned rats were also able to learn a simultaneous discrimination between different digging media in different pots (Experiment 4) and to select a single rewarded location in a cross-maze (place/direction discrimination, Experiment 5). In contrast, the same lesions impaired the ability to learn to eat food in one location but not eat in a second location (Experiment 3). This impairment was, however, selective as the no-go trials seemed particularly affected, a pattern also seen after anterior thalamic lesions [23]. This no-go deficit is consistent with previous descriptions of a failure to withhold trained responses after fornix or hippocampal lesions (e.g., [18,42,43]). In addition, each digging pot location in the go/no-go discrimination was approached from one of two directions. This feature enhances task difficulty and is more strongly associated with go/no-go place deficits after hippocampal and anterior thalamic lesions [13,23], possibly because it creates greater stimulus ambiguity [13,28].

In view of the relatively mild deficits in Experiments 2 and 4, it was valuable to confirm that the fornix lesions caused marked deficits on T-maze place alternation (Experiment 6). Similar deficits have been repeatedly observed on this task after fornix lesions [8,12,41,54,84,87], as well as after hippocampal and anterior thalamic lesions [5,8,9,12,15,32,38,39]. Nevertheless, by the end of T-maze alternation training, the performance levels of the Fornix rats appeared to almost match those of the control group (Fig. 9). It might be supposed that this improvement could reflect a reliance on egocentric (body turn) cues, but previous research has shown how normal rats struggle to use this type of information for delayed alternation [14,52]. Furthermore, the fornix lesion deficit was immediately reinstated by a spatial alternation procedure that excluded the use of intra-maze cues but not body turn cues (adjacent T-mazes, Experiment 7). As intra-maze cues can aid alternation performance [20,22], their use may explain the ability of the rats with fornix lesions to improve their alternation performance during Experiment 6.

The spatial alternation deficit contrasted with the spared ability of the rats with fornix lesions to learn a place/direction discrimination in a cross-maze (Experiment 5). As the cross-maze was rotated between every trial it is most unlikely that intra-maze cues could have assisted. Instead, the constant rewarded direction makes it possible that the head direction system contributed to performance. This system, which provides compass-like signals [69], should be largely spared by fornix lesions as the relevant projections from head direction cells in the anterodorsal thalamic nucleus [34] to the hippocampal formation would involve the cingulum bundle [21], while many of the projections from the head direction areas in the hippocampal formation (the postsubiculum and presubiculum) to the anterodorsal thalamic nucleus use a non-fornical route that relies on the internal capsule [73,74]. The finding that lesions of the anterior dorsal thalamic nucleus are far more disruptive to hippocampal head direction signals than vice versa [30] supports the notion that these spared thalamic efferents could still aid performance after fornix lesions. A similar explanation may partially explain the relatively preserved spatial biconditional task as the two locations could also be distinguished by their principal directions of travel (see Fig. 1).

Experiment 8 examined the ability of rats to use the relative position of particular walls in a square water-maze to identify a specific corner. The fornix lesions impaired spatial learning, as shown by the critical first probe trial (the first time the rats could swim to the escape location). Although the fornix lesions diminished the preference for the correct corner, the rats could still distinguish the correct corner from its mirror-image counterpart (see Fig. 10). This pattern of deficits was repeated in a subsequent transfer test (Two striped walls). Throughout this experiment, extra-maze cues were nullified by rotating the square water-maze between trials and by having a curtain around the apparatus. In addition, the rats learnt the task passively, i.e., no active swim trials were given prior to the first probe test, in order to preclude unwanted solutions such as swimming to the striped wall and then turning right (or left) (see [29]). Consequently, the task requires that the spatial disposition of common elements (e.g., corners composed of both white and striped walls) are discriminated, an ability that lies at the heart of the ‘cognitive map’ hypothesis [51]. Anterior thalamic lesions produce a rather similar set of deficits to those found after fornix lesions as they reduce preference for the escape location, although that location is recognised once it has been reached [26]. Unlike the present fornix lesions, anterior thalamic lesions also increased the latency to first reach the correct corner [26]. Hippocampal lesions, meanwhile, appear even more disruptive as choice performance between mirror-image corners is reduced to chance levels [37]. A refinement for a future study might be to repeat this final experiment in naïve rats with fornix lesions, to ensure that transfer effects from the previous spatial tasks did not contribute to the pattern of results.

When taken together, the present study highlights how the effects of fornix transection in rats are relatively mild on some tasks that depend on the integrity of the anterior thalamic nuclei (Experiment 2), yet cause more comparable deficits on other tasks that also rely on these same thalamic nuclei (Experiments 3, 6, 8). In explaining the present results it should be noted that the lesions were confined to the target tract, i.e., in the large majority of cases there was no additional damage to the corpus callosum or stria medullaris, while the thalamus was typically spared. The selectivity of the surgeries raises the question of whether inadvertently spared fornical fibres prevented a more severe biconditional deficit. It is difficult to make complete fornix transections that do not invade other structures. It is, therefore, relevant that complete fornix disconnections, which are more likely to encroach on adjacent areas, can still spare spatial biconditional learning [24,41,60,62].

The present results point to a complex relationship between the hippocampus and the anterior thalamic nuclei that does not simply reflect their fornical interconnections (see also [42,84,78]). One plausible explanation for the relative sparing seen after fornix lesions concerns the involvement of non-fornical routes from the hippocampal formation to the thalamus. Both the presubiculum and postsubiculum have direct non-fornical projections to the lateral dorsal nucleus, as well as to the anterodorsal nucleus [73,74], thalamic sites that are important for spatial learning [71,72,89]. There may also be non-fornical inputs from the presubiculum and postsubiculum to the dorsal anteroventral nucleus [73,74]. The routes of these various connections increase the likelihood that the head direction system [69] remains functional after fornix damage.

To these direct connections can be added indirect pathways. Of particular interest are the connections via the retrosplenial cortex that link the hippocampal formation with the anterior thalamic nuclei [75–77,82]. Rats with fornix lesions were only severely impaired on a spatial biconditional task when the retrosplenial cortex pathways were also damaged [25], whereas damage to the fornix or to the retrosplenial cortex alone led to little or no deficit [24,56]. The possibility that this indirect retrosplenial pathway can support spatial learning is further strengthened by the results of crossed-lesion disconnection studies involving the retrosplenial cortex with the hippocampus, and the retrosplenial cortex with the anterior thalamic nuclei [57].

There remain other explanations for the present results. Like head direction signals, additional information supporting the spatial biconditional task could originate from the anterior thalamic nuclei to reach the hippocampal formation, without prior involvement of the hippocampus [45,78,83]. Such influences need not involve the fornix [21,55]. Consistent with this view is growing evidence that non-hippocampal inputs to the mammillary bodies, which in turn project to the anterior thalamic nuclei, are critical for a range of spatial functions [78–80,83]. A difficulty with this particular account for explaining the relatively spared spatial biconditional learning seen after fornix lesions is that mammillary body damage also has little or no effect on a range of spatial biconditional tasks [64,65,67], i.e., these additional inputs are typically not needed for spatial biconditional tasks.

The present results prompted a review of those occasions when the effects of fornix and anterior thalamic nuclei lesions have been directly compared within the same study. The impact of fornix and anterior thalamic nuclei lesions appear very similar for T-maze alternation [8,12,84,85,87], for an automated delayed nonmatching-to-position task [6], and for complex item-position discriminations with high ambiguity [27]. In contrast, for learning a fixed location in a Morris water-maze ([84,86,87]; but see [58]) or for learning a location defined by the relative positions of long and short walls in a water tank [12], the impact of anterior thalamic damage often appears appreciably greater than that of fornix surgery. To this second list we can presumably add learning spatial biconditional problems [23].

Given that the hippocampus and the anterior thalamic nuclei function interdependently to support a range of spatial learning tasks, including biconditional learning [32,88], it is evident that their linking fornical connections can only be vital for a subset of spatial problems. This subset appears to consist of those tasks that involve multiple locations and multiple potential responses to those same locations. Consequently, these tasks often involve high levels of proactive interference (e.g., spatial alternation, radial-arm maze foraging, delayed nonmatching-to-position), with added demands on response control as the same response can be reinforced or not reinforced depending on current spatial and temporal information (e.g., go/no-go spatial discrimination). In contrast, spared (non-fornical) hippocampal–anterior thalamic pathways appear largely sufficient to support learning when place-response associations remain constant and the demand on response control is reduced by having a simultaneous discrimination (rather than go/no-go choices). A possible exception to this second category is formed by those problems (e.g., Experiment 8) that involve high levels of feature ambiguity due to overlapping, common elements, although the deficits may appear mild ([26–28,41]; but see [12]). It is perhaps important to note that this distinction between fornical and non-fornical dependent learning is not simply related to task difficulty (as measured by trials to acquire or levels of performance). Forced-place alternation in a T-maze (Experiment 6) is learnt extremely rapidly and normal rats often perform close to ceiling levels, yet it is highly sensitive to fornix lesions [8,12,41,53,84,87].

This analysis has throughout focussed on hippocampal interactions via the fornix with the anterior thalamic nuclei. The rationale largely arose from disconnection studies showing how these two sites are interdependent [32,88]. In reality, the fornix connects the hippocampus with many other sites, including the medial prefrontal cortex and cholinergic forebrain [59]. The disconnection of these additional sites from the hippocampus might be expected to add to any observed pattern of spatial deficits, e.g., affecting response control or recency discriminations [31,36,53]. Fornix lesions might, therefore, be expected to produce deficits consistently greater than those observed after anterior thalamic lesions. Surprisingly, this lesion enhancement is typically not reported ([7,8,12,24,41,60,62,84,87]; but see [57]). One possible explanation is that the fornical interactions with sites such as the prefrontal cortex largely duplicate functions supported by the anterior thalamic nuclei [10]. A closely related possibility is that there is an overshadowing effect, such that the failure of spatial information processing after anterior thalamic damage is so catastrophic that it trumps other deficits. To explain the greater disruptive effect of anterior thalamic damage it may be necessary to assume the recruitment of other, non-fornical pathways involving the hippocampal formation, so interlinking certain key sites [73,74] and ameliorating some of the effects fornix lesions would otherwise have on spatial learning. At the same time, some spatial tasks, e.g., those with high interference, cannot be supported by alternative pathways. In such tasks the information guiding the choice is held in working memory (as in T-maze alternation), making it particularly susceptible to interference. In contrast, for the spatial biconditional problem the guiding stimuli are available at the same time as the choice response, making the task far less sensitive to interference. These ideas point to the need for further disconnection studies that investigate the importance of pathways such at the cingulum and internal capsule, which provide alternative thalamic–hippocampal links [24,50,73,74,50,85].

## Conflict of interest statement

The authors have no conflict of interest to declare.

## Figures and Tables

**Table 1:** Summary table depicting testing arrangements and outcomes of fornix lesions on the various biconditional and spatial tasks in the present study (Experiments 1–8). Performance is indicated as being markedly impaired (‘yes’), mildly impaired (‘mild’), or unimpaired (‘no’).

Experiment	Description	Room	Impaired?
Exp 1	Contextual biconditional discrimination	Pre-train A	No
		Test B	
Exp 2	Spatial biconditional discrimination	B	Mild
Exp 3	Digging media discrimination	B	No
Exp 4	Spatial go/no-go discrimination	B	Yes
Exp 5	Place/Direction cross-maze	C	No
Exp 6	Place alternation T-maze	D	Yes
Exp 7	Place alternation two T-mazes	D	Yes
Exp 8	Passive place learning. One wall probe	Pre-train E	Mild
		Test F	
	Passive place learning. Two wall probe	F	Mild
